# Supplementary material for: DNA Specimen Preservation Using DESS and DNA Extraction in Museum Collections
Source: Biology (Basel). 2025 Jun 19;14(6):730. doi: 10.3390/biology14060730 (PMC12189647; doi:10.3390/biology14060730)
Supplement: Supplementary file 1 [file biology-14-00730-s001.zip › biology-3588417-supplementary/biology-3588417-supplementary 1/biology-3588417-supplementary/FileS1.pdf]

Extraction of meiofauna and meioflora from aquatic environmental samples

Daisuke Shimada[1]

[1]College of Economics, Nihon University

Daisuke Shimada: Center for Molecular Biodiversity Research, National Museum of Nature and Science

Daisuke Shimada

College of Economics, Nihon University, Center for Molecular...

Protocol Info: Daisuke Shimada . Extraction of meiofauna and meioflora from aquatic environmental samples. **protocols.io** **https://protocols.io/view/extraction-of-meiofauna-and-meioflora-from-aquatic-ceyutfww**

Created: August 08, 2022

Last Modified: June 18, 2024

Protocol Integer ID: 68340

Keywords: meiobenthos, specimen, field method, DESS, molecular, morphology

Funders Acknowledgement:
JSPS KAKENHI
Grant ID: JP21K06299

Abstract

Meiofauna and microalgae are benthic communities of small animals and algae, respectively, that can pass through a 1-mm sieve but are retained in a 32-μm sieve. This simplified protocol collects meiofauna and microalgae living in or attached to aquatic substrates such as sediments, seagrasses, seaweeds, and sessile animals. Animals and algae are filtered, killed by heating, fixed with DESS solution, and sorted under a stereomicroscope. The advantage of this method is that both the morphological characteristics and DNA can be preserved for a long period (more than ten years).

Materials

Filter

Nylon mesh (32 or 63 μm)
Wide-mouth screw-cap plastic bottle (500 or 1,000 ml)
Rubber bands

DESS solution

Ethylenediamine-N,N,N',N'-tetraacetic acid disodium salt dihydrate (2Na-EDTA)
Dimethyl sulfoxide (DMSO)
Sodium chloride (NaCl)
Distilled water (DW)

Irwin loop

Stainless steel wire (0.2–0.3 mm diameter)

Sampling

1 Collect substrates (sediments, seagrasses, seaweeds, sessile animals, etc.) by hand or by using sampling devices.

2 Transport entire substrates to the laboratory at low temperature or immediately filter and fix the living organisms in the field by following the steps below. If performing this step is difficult, substrates should be frozen or stored in the DESS solution, despite the risk of injury to organisms.

Filtration

3 Transfer 100–500 ml of substrate into a bucket and wash the substrate well with approximately 10 times volume of tap water. If the substrate is mud, reduce the substrate volume to 50 ml or less. For fragile animals, such as turbellarians, use 3.5% $MgCl_2$ instead of tap water and stir gently. Wait for 10 min or more and stir again gently.

References: Hulings & Gray (1971); Pfannkuche & Thirl (1988); Giere (2009)

CITATION

Giere O (2009). Sampling and processing meiofauna. In: Giere O. Meinbeothology: the microscopic motile fauna of aquatic sediments. Second edition. Berlin; Springer, pp. 63–86.
LINK
https://doi.org/10.1007/978-3-540-68661-3_3

CITATION

Hulings NC, Gray JS (1971). A manual for the study of meiofauna. Smithsonian Contribution of Zoology. 78: 1–83.
LINK
https://doi.org/10.5479/si.00810282.78

CITATION

Pfannkuche O, Thiel H (1988). Sample processing. In: Higgins RP, Thiel H, editors. Introduction to the study of meiofauna. Washington, D.C. & London; Smithonian Institute Press.

4 Immediately pass the supernatants through a 10–100 µm (usually 32 or 63 µm) nylon mesh filter. Smaller mesh sizes can collect more organisms but are easily clogged with debris.

4.1 **How to make a filter**

Remove the bottom from a 500 or 1,000 ml wide-mouth screw-cap plastic bottle and cut a piece of nylon mesh larger than the mouth of the bottle.

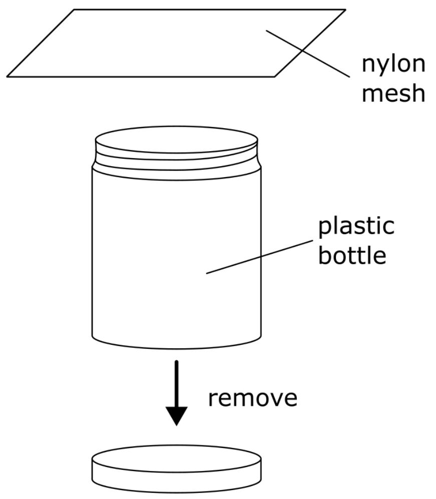

How to make a filter.

4.2 Place the mesh over the mouth of the bottle and fasten tightly with rubber bands.

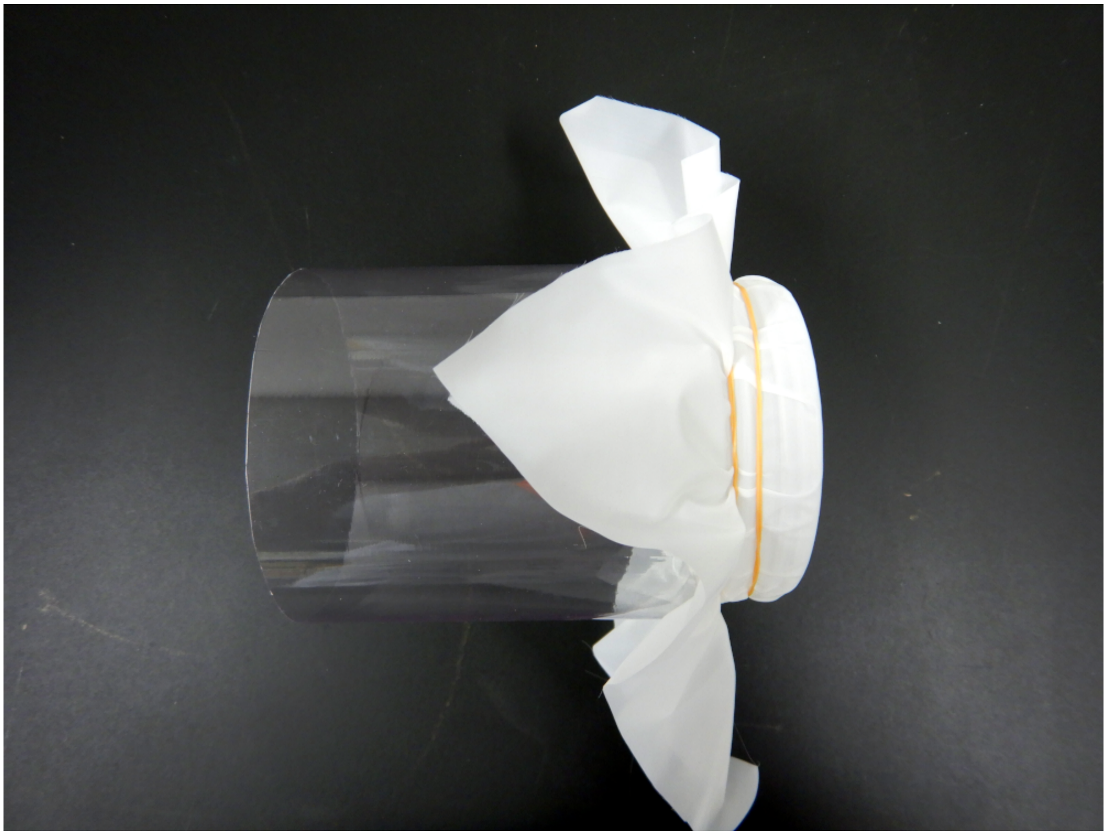

A completed filiter.

5 Repeat steps 3 and 4 once or twice.

6 Carefully remove the nylon mesh from the bottle, turn over, and wash in a Petri dish with 20 ml of filtered seawater. When handling a large substrate amounts or when the mesh appears clogged, the collected material can be transferred to a Petri dish with a dropper after each filtration.

7 Transfer the whole seawater with collected material to a 25–50 ml sampling bottle. If necessary, divide into several bottles.

8 Wait 10 min for the organisms to settle at the bottom and remove as much of the supernatant as possible with a dropper.

Fixation

9 Heat the sampling bottle in 80–90°C water for 2 min to kill the organisms and to inactivate the DNase. Several animals, especially nematodes, straighten when killed by heating. When looking for smaller animals, such as tardigrades, which are easier to find when they are alive, the order of heat killing, fixation, and sorting can be switched.

10 Add 5 to 10 times the volume of DESS solution to the collected material and mix well. The sample can be stored in this state for a long period (more than 10 years).

Reference: Yoder et al. (2006)

CITATION

Yoder M, Tandingan De Ley I, King IW, Mundo-Ocampo M, Mann J, Blaxter M, Poiras L, De Ley P (2006). DESS: a versatile solution for preserving morphology and extractable DNA of nematodes. Nematology. 8: 367–376.

LINK

https://doi.org/10.1163/156854106778493448

10.1 **How to make 1 liter of DESS solution**

Dissolve 93.0 g of 2Na-EDTA in 400 ml of DW by stirring while gradually adding NaOH. Adjust pH to 7.5–8.0 and add DW until the total volume reaches 500 ml (= 0.5M EDTA).

*Buying a pre-conditioned EDTA solution is less time-consuming (but more expensive).

10.2 Mix 500 ml of 0.5 M EDTA with 200 ml (or 200 g) of DMSO and add DW until a final volume of 1,000 ml.

10.3 Add a saturated amount of NaCl and stir well (precipitation of NaCl is not a problem).

Sorting

11 Transfer the sample to a Petri dish. Pick up organisms from the collected material using an Irwin loop under a stereomicroscope and store in 2.0-ml screw-cap micro tubes of DESS solution. To prevent rusting, rinse the loop with DW after each use.

References: Shirayama (2004); Schram & Davison (2012)

CITATION

Shirayama Y (2004). Kaisan senchu [Marine nematodes]. In: Mamiya Y, Araki M, Ishibashi N, Iwahori H, Ogura N, Kosaka H, et al., editors. Senchugaku Jikkenho [Nematological Experimentation]. Tsukuba: The Japanese Nematological Society; pp. 187–194..

CITATION

Schram MD, Davison PG (2012). Irwin loops—a history and method of constructing homemade loops. Transactions of the Kansas Academy of Science. 115: 35–40.
LINK
https://doi.org/10.1660/062.115.0106

11.1 **How to make an Irwin loop**
Cut a stainless-steel wire (0.2–0.3 mm diameter) into 10 cm and 5 cm pieces.

11.2 Hook the pieces together and twist them using pliers, leaving small loops.

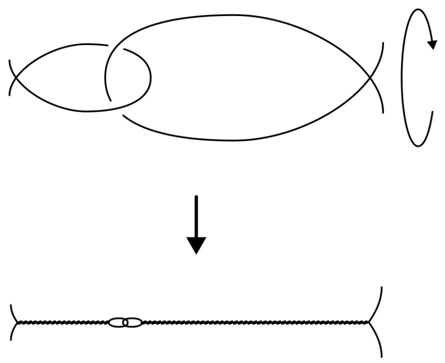

How to make an Irwin loop.

11.3 Remove the shorter piece and fix the longer piece to a suitable handle with water-resistant adhesive.

11.4 Bend the loop part to a manageable angle with pliers.

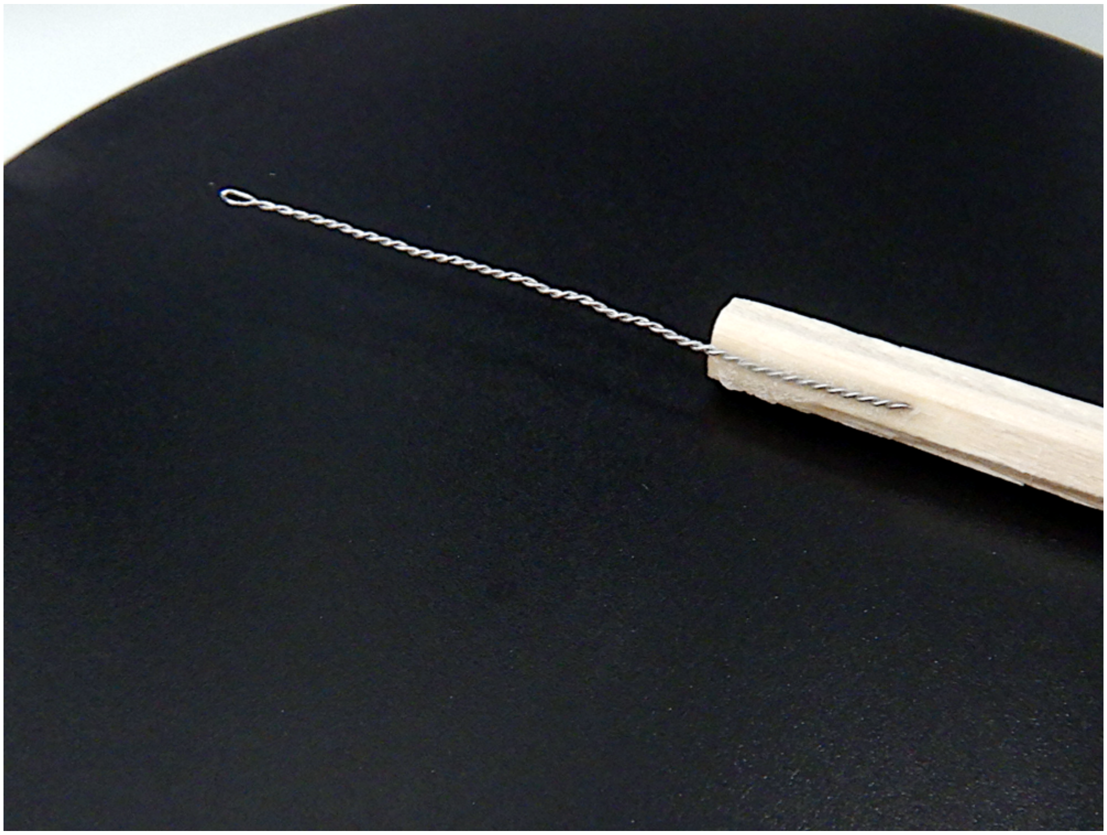

A completed Irwin loop, fixed to a disposable chopstick.

Citations

Step 10

Yoder M, Tandingan De Ley I, King IW, Mundo-Ocampo M, Mann J, Blaxter M, Poiras L, De Ley P. DESS: a versatile solution for preserving morphology and extractable DNA of nematodes
https://doi.org/10.1163/156854106778493448

Step 11

Schram MD, Davison PG. Irwin loops—a history and method of constructing homemade loops
https://doi.org/10.1660/062.115.0106

Step 3

Giere O. Sampling and processing meiofauna
https://doi.org/10.1007/978-3-540-68661-3_3

Step 3

Hulings NC, Gray JS. A manual for the study of meiofauna
https://doi.org/10.5479/si.00810282.78
