# Supplementary material for: DNA Specimen Preservation Using DESS and DNA Extraction in Museum Collections
Source: Biology (Basel). 2025 Jun 19;14(6):730. doi: 10.3390/biology14060730 (PMC12189647; doi:10.3390/biology14060730)
Supplement: Supplementary file 1 [file biology-14-00730-s001.zip › biology-3588417-supplementary/biology-3588417-supplementary 1/biology-3588417-supplementary/TableS1.pdf]

Table S1. Information on Spider species, preservation solutions and leg hardness.

Genus	Species	Sex	Collection Date	Preservation Solution*	Tissue Hardness	Leg length (approx.)
Agelena	*silvatica*	♀	16-IX-2018	A	3	9
Badumna	*longinqua*	♂	14-V-2023	A	3.5	20
Callobius	*hokkaido*	♀	30-IV-2016	A	3	11
Callobius	*hokkaido*	♀	30-IV-2016	A	3	12
Callobius	*yakushimensis*	♀	30-IX-2019	A	3	9
Clubiona	*japonica*	♀	30-IV-2016	A	4	8
Cybaeus	sp.	♀	15-XII-2019	A	3	10
Cybaeus	sp.	♀	13-XI-2020	A	3	10
Cybaeus	sp.	♂	15-XII-2019	A	3.5	10
Lycosidae	sp.	♀	27-XII-2017	A	4	7
Ariadna	*lateralis*	1♀	28-V-2023	B	3	10
Coelotes	*hataensis*	1♀	4-XI-2023	B	2	11
Coelotes	*ibukiensis*	1♀	13-X-2023	B	2	10
Coelotes	*musashiensis*	1♂	22-X-2023	B	3	18
Coelotes	*suruga*	1♀	28-XI-2023	B	2.5	15
Griseidraconarius	*decolor*	1♀	1-XI-2023	B	3	15
Iwogumoa	*interuna*	1♀	15-X-2023	B	2	13
Iwogumoa	*nagasakiensis*	1♀	31-X-2023	B	3	15
Latrodectus	*hasseltii*	1♀	2-XI-2023	B	3.5	15
Spiricoelotes	*zonatus*	1♂	3-XI-2023	B	3	19
Badumna	*insignis*	1♀	18-VI-2023	C	2.5	12
Badumna	*longinqua*	1♀	24-VI-2023	C	3	10
Coelotes	*micado*	1♀	31-X-2023	C	2	13
Griseidraconarius	*decolor*	1♀	1-XI-2023	C	2.5	15
Iwogumoa	*interuna*	1♀	15-X-2023	C	2.5	12
Loxosceles	*rufescens*	1♀	4-XI-2023	C	2	14
Menemerus	*brachygnathus*	1♂	16-V-2023	C	3	9
Sanitubius	*anatolicus*	1♀	10-VI-2023	C	2	11
Tegecoelotes	*corasides*	1♂	30-IX-2023	C	3	13
Xysticus	*croceus*	1♂	16-V-2023	C	2.5	9
Badumna	*longinqua*	1♀	24-VI-2023	D	2.5	10
Coelotes	*kitazawai*	1♀	29-XI-2023	D	2.5	15
Coelotes	*micado*	1♀	31-X-2023	D	2	11
Coelotes	*minobusanus*	1♀	27-XI-2023	D	3	15
Griseidraconarius	*decolor*	1♂	4-XI-2023	D	2.5	13
Iwogumoa	*interuna*	1♀	29-IX-2023	D	1.5	8
Iwogumoa	*interuna*	1♀	15-X-2023	D	1.5	12
Iwogumoa	*nagasakiensis*	1♂	31-X-2023	D	1.5	11
Iwogumoa	*nagasakiensis*	1♀	31-X-2023	D	2	13
Tegecoelotes	*corasides*	1♂	30-IX-2023	D	3	18
Badumna	*longinqua*	1♀ juv.	24-VI-2023	E	5	13
Cladothela	*oculinotata*	1♀	28-V-2023	E	4.5	7
Coelotes	*hiurai*	1♀	29-XII-2016	E	5	10

Coelotes	*kitazawai*	1♂	29-IX-2023	E	4	14
Iwogumoa	*interuna*	1♀	30-IX-2023	E	4	13
Iwogumoa	*interuna*	1♀	15-X-2023	E	4	10
Marpissa	*pulla*	1♂	27-V-2023	E	5	6
Pardosa	*laura*	1♀	16-V-2023	E	4	10
Plexippoides	*doenitzi*	1♀	16-V-2023	E	5	9
Tegecoelotes	*corasides*	1♂	30-IX-2023	E	4.5	18
Badumna	*insignis*	1♂	18-VI-2023	F	3	10
Coelotes	*micado*	1♀	31-X-2023	F	3.5	12
Coelotes	*musashiensis*	1♀	28-XI-2023	F	3.5	14
Cybaeus	*nipponicus*	1♀	29-XI-2023	F	3.5	15
Cybaeus	*nipponicus*	1♂	30-XI-2023	F	3.5	14
Iwogumoa	*interuna*	1♀	15-X-2023	F	3	11
Iwogumoa	*nagasakiensis*	1♀	31-X-2023	F	3	11
Badumna	*longinqua*	1♀ juv.	24-VI-2023	G	4.5	9
Coelotes	*kitazawai*	1♂	7-X-2023	G	3.5	12
Coelotes	*musashiensis*	1♀	7-X-2023	G	3.5	13
Griseidraconarius	*decolor*	1♀	15-X-2023	G	2	11
Iwogumoa	*interuna*	1♂	29-IX-2023	G	2	10
Iwogumoa	*interuna*	1♀	15-X-2023	G	3	12
Iwogumoa	*nagasakiensis*	1♂	30-X-2023	G	3	14
Marpissa	*milleri*	1♂	1-X-2023	G	3	9
Tegecoelotes	*corasides*	1♂	30-IX-2023	G	3	18
Uroctea	*compactilis*	1♀	29-IX-2023	G	2	11
Aeolocoelotes	*personatus*	1♀	12-X-2023	H	1.5	12
Badumna	*insignis*	1♀	18-VI-2023	H	1.5	13
Badumna	*longinqua*	1♂ juv.	24-VI-2023	H	3	10
Coelotes	*micado*	1♂	30-X-2023	H	2	13
Coelotes	*musashiensis*	1♀	29-IX-2023	H	2	12
Coelotes	*musashiensis*	1♂	27-XI-2023	H	2	13
Cybaeus	sp.	1♂	30-IX-2023	H	1.5	10
Griseidraconarius	*decolor*	1♀	1-XI-2023	H	2.5	15
Iwogumoa	*interuna*	1♀	15-X-2023	H	1	9
Tegecoelotes	*corasides*	1♀	27-XI-2023	H	1.5	15

* Preservation solutions: A:100% DESS; B: 75% DESS; B: 75% DESS; C: 50% DESS; D: 25% DESS; E: anhydrous ethanol; F:70% ethanol; G:70% ethanol (10mM EDTA); H:70% ethanol (1mM EDTA)
